# Supplementary material for: Natural Fiber-Reinforced Composite Incorporated with Anhydride-Cured Epoxidized Linseed-Oil Resin and Atmospheric Pressure Plasma-Treated Flax Fibers
Source: Materials (Basel). 2024 Aug 28;17(17):4244. doi: 10.3390/ma17174244 (PMC11396233; doi:10.3390/ma17174244)
Supplement: Supplementary file 1 [file materials-17-04244-s001.zip › materials-3149812-supplementary.pdf]

Supplementary Materials

Table S1. Surface energy values of raw flax tape(M1) and non-woven flax (M2), where air plasma is indicated as A and nitrogen as N, ELO – short name for cured resin and the last number shows the number of treatment cycles.

Sample's name	SFE [mN/m]	Polar Part [mN/m]	Dispersive part [mN/m]
M1.0	23.57±6.69	15.7±4.63	7.87±2.05
M1.A.1	34.05±7.75	22.42±5.22	11.63±2.53
M1.A.5	30.29±11.2	19.37±8.03	10.92±3.17
M1.A.10	42.5±7.93	34.1±6.32	8.4±1.62
M1.N.1	35.97±18.27	24.73±13.69	11.24±4.58
M1.N.5	34.28±11.75	22.53±8.74	11.76±3.01
M1.N.10	39.5±10.88	30.99±8.58	8.51±2.3
M2.0	32.86±6.66	22.28±4.45	10.58±2.22
M2.A.1	41.68±4.39	32.53±3.3	9.15±1.09
M2.A.5	41.22±3.5	32.27±2.59	8.95±0.92
M2.A.10	43.96±3.87	36.5±2.9	7.47±0.97
M2.N.1	40.15±4.68	30.9±3.59	9.23±1.09
M2.N.5	41.08±5.34	32.25±4.12	8.82±1.22
M2.N.10	41.48±4.16	34.28±3.31	7.2±0.85
M2.ELO	31.5±4.09	0.01±0.14	31.49±3.95
M2.A.ELO.10	28.78±3.49	13.59±2.14	15.18±1.35

Table S2. Composite processing data.

Name	Precured time [min]	Processing speed [m/min]	Processing time [min]	Average Elastic Modulus [GPa]
C1	0	1 m/min	2.5	18.28±0.32
C2	20	1 m/min	2.5	18.07±2.7
C3	30	1 m/min	2.5	14.77±1.76
C4	40	1 m/min	2.5	18.3±0.2
C5	0	2.5 m/min	1	15.93±1.6
C6	20	2.5 m/min	1	17.65±0.35
C7	30	2.5 m/min	1	15.87±2.75
C8	40	2.5 m/min	1	16.47±0.64
